# Physiological and Transcriptome Analyses Revealed the Mechanism by Which Deferoxamine Promotes Iron Absorption in *Cinnamomum camphora*

**DOI:** 10.3390/ijms23179854

**Published:** 2022-08-30

**Authors:** Wei-Liang Kong, Tong-Yue Wen, Ya-Hui Wang, Xiao-Qin Wu

**Affiliations:** 1 Co-Innovation Center for Sustainable Forestry in Southern China, College of Forestry, Nanjing Forestry University, Nanjing 210037, China; 2 Jiangsu Key Laboratory for Prevention and Management of Invasive Species, Nanjing Forestry University, Nanjing 210037, China

**Keywords:** iron deficiency, siderophores, *Cinnamomum camphora*, deferoxamine, transcriptome

## Abstract

Iron deficiency causes chlorosis and growth inhibition in *Cinnamomum camphora*, an important landscaping tree species. Siderophores produced by plant growth-promoting rhizobacteria have been widely reported to play an indispensable role in plant iron nutrition. However, little to date has been determined about how microbial siderophores promote plant iron absorption. In this study, multidisciplinary approaches, including physiological, biochemical and transcriptome methods, were used to investigate the role of deferoxamine (DFO) in regulating Fe availability in *C. camphora* seedlings. Our results showed that DFO supplementation significantly increased the Fe^2+^ content, SPAD value and ferric-chelate reductase (FCR) activity in plants, suggesting its beneficial effect under Fe deficiency. This DFO-driven amelioration of Fe deficiency was further supported by the improvement of photosynthesis. Intriguingly, DFO treatment activated the metabolic pathway of glutathione (GSH) synthesis, and exogenous spraying reduced glutathione and also alleviated chlorosis in *C. camphora*. In addition, the expression of some Fe acquisition and transport-related genes, including *CcbHLH*, *CcFRO6*, *CcIRT2, CcNramp5*, *CcOPT3* and *CcVIT4*, was significantly upregulated by DFO treatment. Collectively, our data demonstrated an effective, economical and feasible organic iron-complexing agent for iron-deficient camphor trees and provided new insights into the mechanism by which siderophores promote iron absorption in plants.

## 1. Introduction

Iron is a key element in the normal development of plants. Because it is a cofactor of many metabolic pathways, its lack may lead to the interruption of many processes, including respiration or photosynthesis, and may become the cause of subsequent chlorosis [1,2]. Iron is the fourth most abundant element in the Earth’s crust and is abundant in most types of soil [3]. This element exists in two states in aqueous solution: Fe^2+^ and Fe^3+^. However, Fe^3+^ forms are not easily absorbed by plants because they often form insoluble oxides or hydroxides, thus limiting bioavailability [4,5]. It has been estimated that approximately 1/3 of the Earth’s soils have potential iron deficiency; thus, iron deficiency has become another important nutritional barrier for agroforestry production (after nitrogen and phosphorus deficiencies) [6].

In the long process of evolution, plants have developed two strategies to obtain iron. Strategy I involves acidification of the rhizosphere, followed by reduction of Fe^3+^ to Fe^2+^ by the plasma membrane-bound ferric chelate reductase (FCR) and subsequent Fe^2+^ transport into root cells [7]. Strategy II refers to plants that secrete low-molecular-weight plant siderophores to dissolve and bind iron and then transport it to root cells through membrane proteins [1]. However, these strategies are often insufficient to meet the needs of plants, especially those growing in calcareous and alkaline soils [8]. Therefore, when iron is deficient in the soil, it is necessary to provide plants with available iron. A large number of studies have shown that many plant rhizosphere growth-promoting bacteria can produce siderophores that can form chelates with Fe^3+^ with a very high stability coefficient, which can improve the iron nutrition of plants, so this absorption pathway has been classified as the third mechanism of iron absorption by plants [9,10,11]. Wang et al. conducted a hydroponic experiment with purified siderophores and found that the growth indexes (plant height, root length, leaf length and fresh weight) of cucumber seedlings supplemented with insoluble Fe_2_O_3_ and siderophores were the highest, indicating that siderophores played an important role in the transformation of insoluble iron into plant available iron [12]. Peas, wheat and rape inoculated with siderophore-producing bacteria showed higher growth and yield [13,14,15]. Pyoverdine produced by *Pseudomonas fluorescens* can induce the physiological response, iron absorption and chlorophyll content of tomato and can regulate the expression of *SlFRO1* and *SlIRT1* in tomato, thus improving the iron nutrition of tomato plants [16].

As of the present, there have been many studies on the effects of inoculating siderophore-producing bacteria on plant iron nutrition, but little is known about the mechanism by which microbial siderophores regulate plant iron metabolism. Some studies have shown that the Fe^3+^ in the siderophores−Fe^3+^ complex was not easily reduced by the root reductase of its host plant, but it could be directly absorbed, transformed and utilized by the plant through endocytosis [17], while other researchers have insisted that the microbial siderophores only acts as a medium. After entering the root cell membrane, the complex will first be reduced to the ferrous form by the plant’s own ferric chelate reductase (FCR) and then transported to the root by the Fe^2+^ transporter [18,19]. Therefore, it was deemed necessary to use new technical means to carry out in−depth research on this issue.

Transcriptome sequencing refers to the use of second generation high−throughput sequencing technology for cDNA sequencing, which can comprehensively and quickly obtain almost all transcripts of a specific organ or tissue of a species in a certain state [20,21,22]. In recent years, RNA−seq technology has been used to analyse the molecular mechanism of plant tolerance to iron deficiency stress, and key *FRO*, *IRT* and TF family genes have been found in many plant species, such as *Arabidopsis thaliana*, pea, cucumber and tomato [23,24,25]. *Cinnamomum camphora* is a common tree species in urban landscaping. Iron deficiency yellowing seriously affects its local economic and ecological benefits, and the lack of genome resources for *C. camphora* greatly hinders the further exploration of the mechanism of low−iron tolerance of *C. camphora* in the rhizosphere of saline−alkali soil [26].

Deferoxamine (DFO) is the first siderophores isolated from *Streptomyces* and the most effective iron fertilizer known thus far [27,28]. It has been widely used as an iron source for crops such as peanut, cucumber, tobacco and wheat [29,30,31]. In the early stage, we found that *Rahnella aquatilis* JZ-GX1 could also produce deferoxamine, which was related to the relief of iron deficiency etiolation of *C. camphora* [32]. However, the mechanism by which deferoxamine promotes the iron absorption of camphor remains unclear, and studies on its use as an iron fertilizer for woody plants have not been reported. By combining physiological characteristics with comparative transcriptomes, this study aimed to (1) explore the absorption and utilization of the siderophores−Fe^3+^ complex by plants, (2) identify genes related to camphor iron uptake and (3) guide breeders in carrying out germplasm innovation by transferring transgenic technology into physiologically etiolated camphor plants.

## 2. Results

### 2.1. Deferoxamine Promoted the Iron Absorption of C. camphora

**Figure 1:**
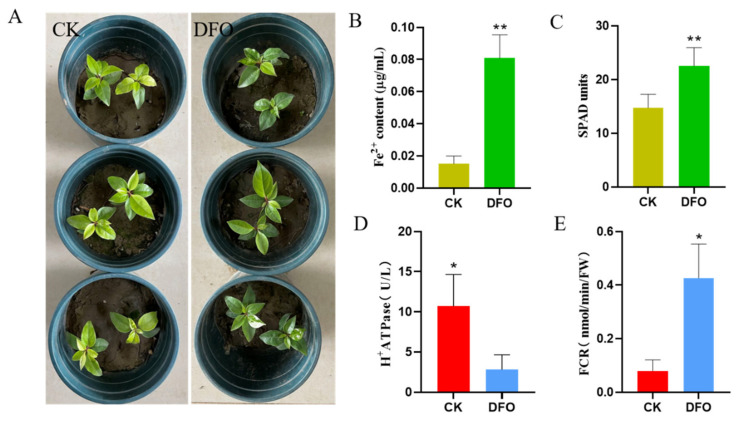
Effects of deferoxamine on the growth of *C. camphora* under iron deficiency. (**A**) Plant phenotype, (**B**) Fe^2+^ content, (**C**) SPAD value, (**D**) H^+^ ATPase and (**E**) ferric chelate reductase. Bars represent the standard error (*n* ≥ 3). * and ** represent significant differences at *p* < 0.05 and *p* < 0.01 (*t*-test), respectively.

The young leaves of *C. camphora* planted in alkaline soil showed a typical iron deficiency yellowing phenomenon, but the addition of DFO eliminated this phenomenon, and the leaves were dark green (Figure 1A). The Fe^2+^ and chlorophyll concentrations in leaves were 436.42% and 52.83% higher than those of CK (Figure 1B,C).

Next, we determined two key enzymes in the process of iron absorption in plants. Compared with CK, camphor roots supplemented with DFO had lower H^+^ ATPase and higher FCR activity (Figure 1D,E). This indicated that the addition of exogenous iron sources reduced the sensitivity of the plants to iron deficiency, while the DFO−Fe^3+^ chelates absorbed by plants needed to be reduced by FCR.

### 2.2. The Whole Transcriptional Response Spectrum of C. camphora to Deferrioxamine

**Figure 2:**
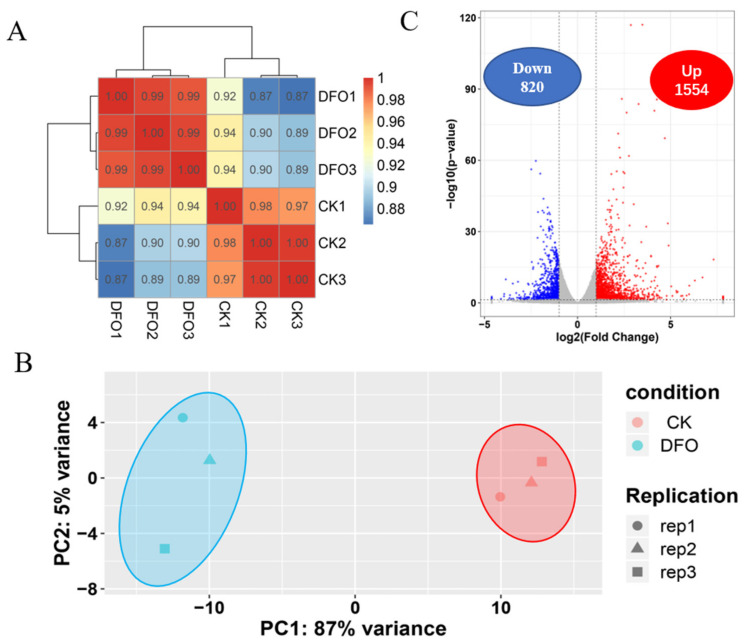
Global analysis of the transcriptome characteristics of *C. camphora* roots with and without deferoxamine. (**A**) Pearson distribution, (**B**) PCA diagram and (**C**) volcano plot.

To elucidate the molecular mechanism by which deferoxamine promotes iron uptake in camphor trees, the whole genome expression profile of the camphor tree root system was screened by RNA−seq. Based on the Pearson correlation coefficient, it was found that the repeatability of the sample was good, and the correlation heatmap was used to present the results (Figure 2A). The repeatability of the samples was further confirmed by the PCA results, and the two groups of samples could be clearly distinguished (Figure 2B). Differential analysis showed that, among the 2374 differentially−expressed genes (DEGs), compared with the treatment without DFO, 1554 genes were upregulated and 820 genes were downregulated in the plants treated with DFO. These results were presented as a volcano plot to observe the overall distribution of the data (Figure 2C).

### 2.3. Analysis of the GO and KEGG Enrichment Pathways of DFO-Induced Transcriptional Changes in C. camphora

**Figure 3:**
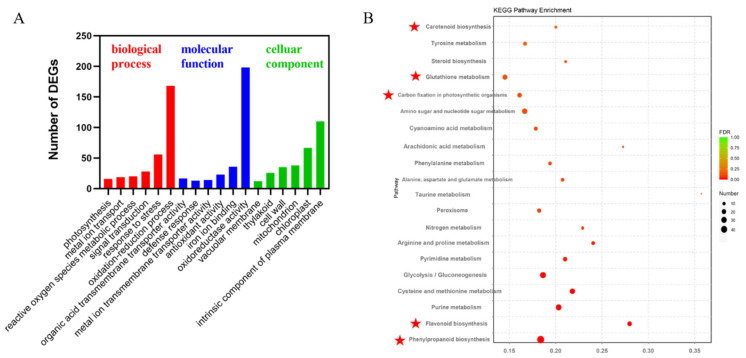
GO (**A**) and KEGG (**B**) analyses of the transcriptional changes of DEGs induced by DFO in *C. camphora.* The circle colour and size indicate the *p*-value and gene number, respectively.

In biology, different genes coordinate and perform their biological functions, and pathway analysis can be used to show the biological functions of genes. Through the functional enrichment analysis of GO, the functional classification and prediction of DEGs in the control group and DFO treatment group were carried out. These DEGs were further divided into three categories: biological processes (BP), cellular components (CC) and molecular functions (MF). In BP, the DEGs participated in the redox reaction, pressure response, signal transduction and active oxygen metabolism. In MF, iron ion binding, antioxidant activity, metal ion transport and the defensive response were activated. In CC, most DEGs were related to the chloroplast, mitochondria, thylakoid and tonoplast (Figure 3A). KEGG enrichment analysis showed that the DEGs mainly functioned in carotenoid biosynthesis, glutathione metabolism, carbon fixation in photosynthetic organisms, flavonoids and phenylpropane biosynthesis (Figure 3B).

### 2.4. DFO Regulates the Expression of Photosynthesis-Related Genes in C. camphora

**Figure 4:**
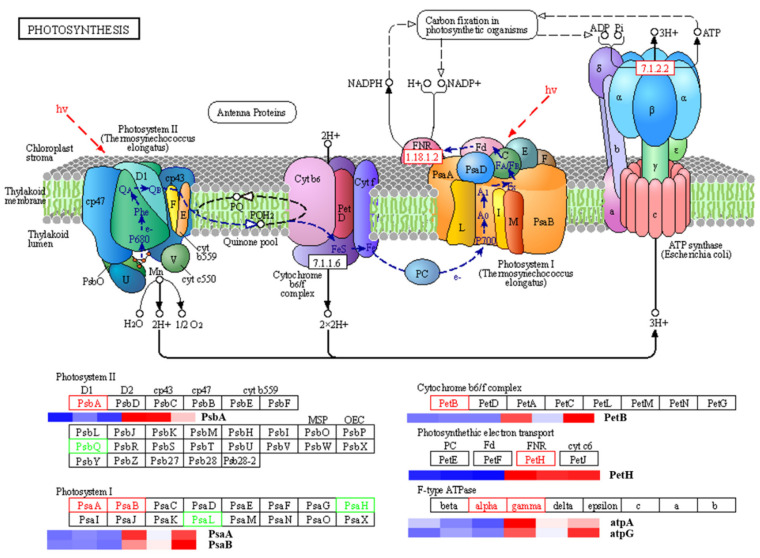
Summary of DEGs in the photosynthesis pathway in *C. camphora* treated with DFO. The bar represents the scale of the expression levels for each gene (FPKM) in the different treatments, as indicated by blue/red rectangles. Genes in blue show DFO treatment (upregulation), and those in red show CK treatment (downregulation).

In the photosynthetic pathway, compared with CK, the expression of *PsbA*, a key light regulatory gene related to the PS II system, and the subunit proteins *PsaA* and *PsaB*, which encoded the PSI reaction centre, were upregulated in the DFO treatment. The expression of *PetB*, a protein related to the cytochrome b6f complex, and ferredoxin-NADP^+^ reductase was upregulated during photosynthetic electron transport. In addition, there were 8 types of genes encoding F−type ATP enzyme-related proteins, of which 2 types of genes were upregulated: alpha (F−type H^+^ transport ATPase−alpha subunit) and gamma (F−type H^+^ transport ATPase−gamma subunit) (Figure 4). The above results showed that the application of DFO could effectively promote the light response efficiency of *C. camphora*.

### 2.5. Reduced Glutathione (GSH) Contributes to DFO Alleviating Iron Deficiency Etiolation of C. camphora

**Figure 5:**
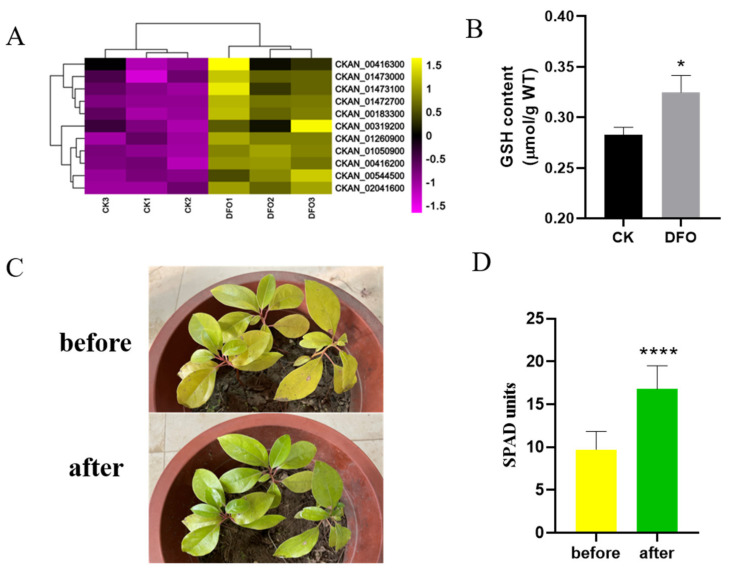
GSH alleviates the DFO−mediated iron deficiency response in *C. camphora*. (**A**) Gene expression analysis of the GSH metabolic pathway with and without DFO supplementation, (**B**) comparison of the endogenous GSH content, (**C**,**D**) phenotype and SPAD value of *C. camphora* before and after exogenous spraying of 1 μM reduced GSH. Bars represent the standard error (*n* ≥ 3). * and **** represent significant differences at *p* < 0.05 and *p* < 0.0001 (*t*-test), respectively.

According to the results of KEGG enrichment analysis, we analysed the genes that changed the glutathione metabolism pathway in *C. camphora* treated with DFO and found that 11 genes, i.e., eight glutathione S−transferase genes, two glutathione peroxidase genes and one glutathione hydrolase gene, were upregulated (Figure 5A). By detecting the content of endogenous GSH in *C. camphora*, it was found that, compared with CK, it increased by 14.94% (Figure 5B). Then, 10 days after exogenous GSH treatment, we observed that the etiolated camphor plants showed an obvious green colour (Figure 5C), and there was a significant difference in the SPAD value before and after application (Figure 5D). These results showed that GSH participated in the iron deficiency etiolation of *C. camphora* alleviated by DFO.

### 2.6. Transcription Factor Family Analysis of C. camphora Caused by DFO

**Figure 6:**
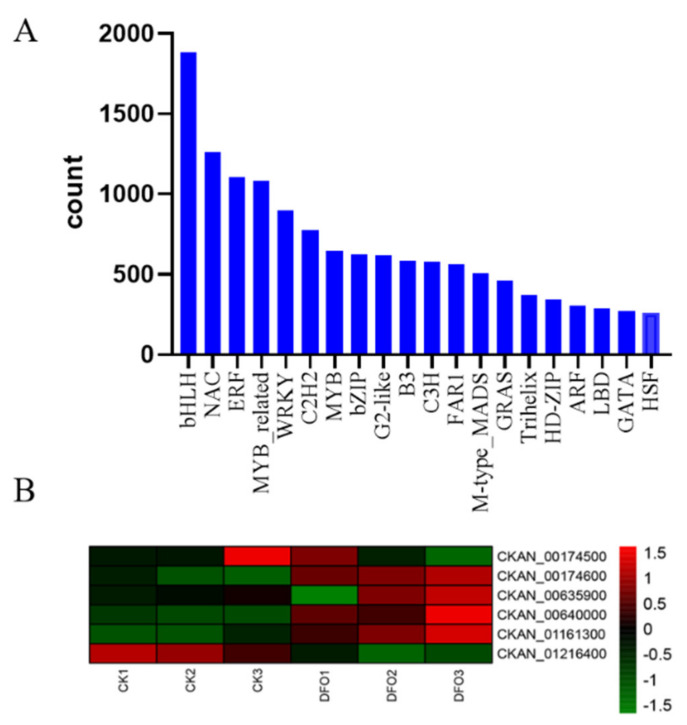
Transcription factor family analysis of DFO−induced transcriptional changes in *C. camphora*. (**A**) Quantitative statistics of transcription factor proteins and (**B**) the expression profile of transcription factors related to iron transport regulation. The bar represents the scale of the expression levels for each gene (FPKM) in the different treatments, as indicated by green/red rectangles. Genes in red show upregulation, and those in green show downregulation.

The process of transcription initiation in eukaryotes is very complex and often requires the assistance of a variety of protein factors. In view of the importance of transcription factors in biological processes, information on all transcription factors was summarized from transcriptome data. A total of 58 transcription factor family proteins were found in the control and DFO-treated camphor roots, among which the highest number of transcription factors was bHLH (1886), followed by NAC (1260), ERF (1104), MYB_related (1081), WRKY (897), C2H2 (774), MYB (649) and bZIP (625) (Figure 6A). To identify the transcription factors related to the regulation of iron transport, we screened six genes in the bHLH family and found that, compared with CK, DFO treatment significantly upregulated the other five iron deficiency-induced transcription factor proteins (FIT), except the CKAN_01216400 gene (Figure 5B).

### 2.7. RT–qPCR Verification of Transcriptome Data with and without DFO Camphor Roots

**Figure 7:**
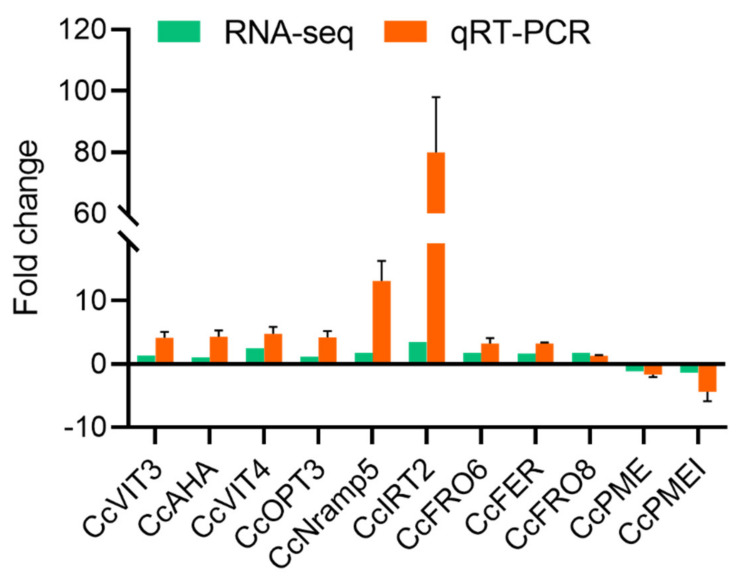
RT−qPCR verification of transcriptome data for camphor tree roots with and without DFO.

To verify the reliability of the RNA−seq data, RT−qPCR was performed on randomly selected genes. The results showed that, after the addition of DFO, 11 selected genes showed differential expression between the two treatments, of which 9 genes were upregulated, the differential expression ratio of the *CcIRT2* and *CcNramp5* genes was the highest and two genes (*CcPME* and *CcPMEI*) were downregulated (Figure 7). The expression pattern of the RT−qPCR results was roughly consistent with the trend of the transcriptome data, which confirmed the reliability of the transcriptome sequencing results.

## 3. Discussion

A common strategy for the treatment of iron deficiency is to apply a variety of inorganic and organic iron fertilizers [33]. Compared with inorganic iron salts, iron chelates have been highly recommended for the treatment of iron deficiency yellowing because Fe(II) oxidizes rapidly when exposed to air, while chelated iron is more easily transferred within plants [34,35]. Commonly used organic iron fertilizers include FeEDDHA, FeDTPA, and FeEDTA [36,37,38]. Excessive use of synthetic chelates such as EDTA and EDDHA may cause concern about environmental pollution because they are either nondegradable or poorly biodegradable [39]. Therefore, it has become necessary to find and develop environmentally safe organic iron-complexing agents to prevent iron deficiency yellowing of plants. Deferoxamine is a siderophores secreted by microorganisms and has been widely used in crop production due to its high biosafety [40,41]. In this study, deferoxamine was applied to alleviate the iron deficiency yellowing of *C. camphora*, and it was found that 10 μM DFO played a beneficial role, greatly reducing the application cost and expanding the application range of the product.

Under the action of light, chlorophyll in plants absorbs carbon dioxide from the air, combines the water absorbed by roots to synthesize carbohydrates and releases oxygen necessary for human survival [42]. Many iron−containing compounds in plants are involved in photosynthesis. These include the cytochrome oxidase complex and ferredoxin [43]. When plants are deficient in iron, the photosynthetic rate decreases, which leads to the disintegration of thylakoids, which inhibits photosynthesis. At the same time, the carotenoid and ferredoxin contents in the chloroplast pigment protein complex also decrease, which inhibits the synthesis of chloroplast precursors and reduces the chlorophyll content [44]. In this study, the active Fe and chlorophyll contents of camphor trees supplemented with deferoxamine increased significantly. Through GO and KEGG enrichment analyses, it was found that most of the differentially-expressed genes were involved in cellular components related to chloroplasts, thylakoids and mitochondria. Carbon fixation and the carotenoid biosynthesis pathway were activated, indicating that the application of deferoxamine promoted the photosynthesis and growth of camphor trees.

Glutathione (GSH) is an important antioxidant that exists widely in plants and is involved in the scavenging of excess reactive oxygen free radicals, the reduction of peroxides, redox-sensitive signal transduction, complexation with hetero toxic substances, the regulation of plant growth and development and resistance to various stresses (heavy metals, drought, salt stress, and bacterial infection) during the process of cell metabolism [45]. When plants suffer from iron deficiency stress, the level of reactive oxygen species (ROS) increases, resulting in membrane lipid peroxidation [46]. As an important cofactor in the synthesis of many enzymes, iron deficiency leads to a decrease in antioxidant activities, such as POD and CAT, and causes plant cells to accumulate more H_2_O_2_ [47]. Results showed that the application of deferoxamine induced the synthesis of glutathione through a nonenzymatic system, which activated the metabolic pathways of antioxidant activity, active oxygen metabolism and the pressure response, thus improving the antioxidant level of iron-deficient *C. camphora*.

Iron uptake, transport and homeostasis in plants are precisely regulated by transcription factors at different levels, such as the transcriptional level, translation level and posttranslational modification, to avoid or reduce the adverse effects of iron deficiency and iron excess on plant growth and development [48]. At least 16 bHLH transcription factors form complex regulatory networks that are closely involved in the regulation of plant iron homeostasis [49]. Based on the FER tomato mutant, Ling et al. isolated and identified the first bHLH transcription factor FER (Fer-like iron deficiency-induced transcription factor) involved in the regulation of iron uptake by map-based cloning [50]. The FER-deficient tomato mutant could not induce the expression of the Fe^3+^ reductase gene *LeFRO1* or the Fe^2+^ transporter gene *LeIRT1*. Subsequently, Bauer et al. cloned the homologous gene *AtbHLH29*/*FIT* of FER in *Arabidopsis thaliana* and proved that it was a key transcription factor responsible for regulating the expressions of *FRO2* and *IRT1* [51]. In this study, DFO triggered the expressions of five bHLH transcription factors, CKAN_00174500, CKAN_00174600, CKAN_00635900, CKAN_00640000 and CKAN_ 01161300, while inhibiting the expression of CKAN_01216400, indicating that *C. camphora*, similar to other dicotyledons, has a set of core components regulated by iron deficiency and that different components perform different functions.

In addition to bHLH transcription factors, downstream functional genes play an important role in plant absorption and transport. In Fe uptake, *FRO* is involved in the reduction of Fe^3+^ to Fe^2+^. *IRT1* is the main transporter involved in Fe^2+^ uptake by plants and exists in almost all plants. As such, the study of *IRT1* is of great significance in efforts to understand the mechanism of iron absorption and maintain iron homeostasis [52,53]. It has been determined that the NRAMP family displays different functions in different plant species. Among the six NRAMP genes in *A. thaliana*, *AtNRAMPl−4* is related to metal ion transport. Overexpression of *AtNRAMP1/2* enhances the tolerance of plants to high-speed iron [54]. The VIT gene family is ubiquitous in monocotyledons, dicotyledons, bacteria and fungi. This family encodes a transporter that mediates the transport of Fe^2+^ into vacuoles [55]. OPT plays an important role in the process of material transfer in plants [56]. Therefore, in this study, the expression levels of 9 genes related to iron metabolism were analysed, and the results showed that these genes were significantly upregulated by DFO.

Pectin is one of the main components of the plant cell wall and the main binding site of cations. Pectin methyl esterase (PME) is commonly found in different tissues and organs of higher plants, such as the roots, stems, leaves, and fruits, and it plays an important role in cell wall composition and degradation, root tip extension and disease resistance [57]. Under the condition of iron deficiency, the PME activity of plant roots was enhanced, which made pectin produce more free carboxyl groups (that is, cationic adsorption sites) and increased the binding ability to iron [58]. Plant pectin methyl esterase inhibitor binds to pectin methyl esterase to form a reversible complex at a 1:1 ratio to inhibit its activity [59]. The results of transcriptome analysis showed that the expression levels of pectin methyl esterase and the pectin methyl ester inhibitor in roots treated with DFO were lower than those of CK, indicating that DFO may decrease pectin methyl esterase activity and iron ion fixation by inhibiting the expression of pectin methyl esterase. DFO resulted in higher amounts of iron transported to the aboveground plant parts through long distance transport and, finally, increased the content of active iron in the leaves, which turned the leaves green.

## 4. Materials and Methods

### 4.1. Plant Material and Growth Conditions

Three-month-old *C. camphora* seedlings were acquired from Guilin, Guangxi, and planted in 15-cm diameter × 10-cm high plastic pots, with two seedlings per pot, and 500 g of soil. Saline-alkali soil samples were collected from uncultivated soil at Xuzhou. The soil properties were as follows: pH: 8.2, organic matter content: 12.45 g/kg, nitrate nitrogen: 12.50 mg/kg, available phosphorus: 10.20 mg/kg, available potassium: 1.41 mg/kg and effective Fe: 6.14 mg/kg.

Deferoxamine was purchased from Rongshide Trading Co., Ltd. (Nanjing, China), diluted to 10 μM with distilled water and then poured onto the roots of *C. camphora*. A treatment with only distilled water was used as the control. After one month, the active Fe and physiological indexes of *C. camphora* were determined. All *C. camphora* seedlings were incubated in a growth chamber at 25 °C and 70% relative humidity with a 12-h light/dark cycle. There were 10 pots per treatment, with 2 seedlings in each pot, for a total of 20 seedlings.

### 4.2. SPAD Value

Changes in the chlorophyll content in the *C. camphora* seedlings were measured using a SPAD-502 portable chlorophyll meter (Minolta Camera Co., Ltd., Osaka, Japan). Two true leaves at the top of each plant were selected for determination, and each treatment was repeated five times [60].

### 4.3. Determination of the Endogenous Iron Content

An improved NH_4_F masking method was used for the extraction of Fe^2+^ from the fresh samples. The concentrations of Fe^2+^ in the digested solution were then determined with O-phenanthroline spectrophotometry [61,62].

### 4.4. Assays for H^+^ ATPase and FCR Activities and the Glutathione Content in Plants

To verify whether the expression of key iron absorption genes was consistent with the enzyme activity, H^+^ ATPase and FCR were extracted from plant roots according to the research of Zhang et al. [63] and Arikan et al. [64], and their activities were measured at 450 nm and 562 nm, respectively. The glutathione content was determined according to the instructions of the kit obtained for glutathione measurement (Keming Biotechnology Co., Ltd., Suzhou, China), and the OD was recorded at 412 nm using a spectrophotometer.

### 4.5. RNA-Sequencing (RNA-Seq) Analysis

After transplantation of three-month-old *C. camphora* into saline-alkali soil, 10 µM DFO solution was added to one treatment, and *C. camphora* without DFO served as a control. After 48 h of culture, the control and DFO-treated roots were used to extract total RNA. The quality of the RNA samples was detected using an Agilent 2100 Bioanalyzer (Illumina, San Diego, CA, USA). The raw sequencing data were processed by removing the low-quality reads and were then submitted to the NCBI SRA database (PRJNA857520). Differentially-expressed genes (DEGs) were identified as follows: FDR-adjusted *p* value < 0.05 and fold change > 1.0. Gene direction and functions were annotated based on the Nr annotations. Gene ontology (GO) annotations with the default parameters were analysed by the Blast2 GO program and were clustered into three groups: biological process, cellular component, and molecular function. Furthermore, the identification of differentially-expressed genes (DEGs) between the NI and I libraries was conducted using a rigorous algorithm at a false discovery rate (FDR)-adjusted *p* value < 0.05. GO term 2 was assigned to DEGs based on the above GO annotations. In addition, GO enrichment analysis was performed to search for significantly enriched functional classifications.

### 4.6. Real-Time Quantitative PCR (RT–qPCR)

Total RNA was extracted by a rapid plant RNA extraction kit (Beijing Zoman Biotechnology Co., Ltd., Beijing, China), and genomic DNA contamination in RNA samples was digested by DNase (Promega, Madison, WI, USA). Then, approximately 1000 ng of total RNA was reverse transcribed into first-strand cDNA using a PrimeScript R RT Reagent kit (CAT: 11202ES08; Yeasen, Shanghai, China) following the manufacturer’s instructions [65]. The relative expression levels of *CcFRO6*, *CcFRO8*, *CcIRT2*, *CcVIT3, CcVIT4*, *CcFER*, *CcOPT3*, *CcNramp5*, *CcPME, CcPMEI* and *CcAHA2* were determined, and the *CcEF1α* gene was used as the internal control. The expression levels of related genes were calculated by ABI 7500 software (Applied Biosystems v2.3, Waltham, MA, USA) and the 2^−ΔΔCT^ method [66]. The primers used to amplify these genes are listed in Table 1.

### 4.7. Data Analysis and Processing

Data are presented as the means ± standard error (SE). Statistical differences between treatments were evaluated using independent-samples Student’s *t*-tests in GraphPad Prism 8.0 (GraphPad Software, Inc., San Diego, CA, USA), with * *p* < 0.05, ** *p* < 0.01 and **** *p* < 0.0001 as significant.

## 5. Conclusions

**Figure 8:**
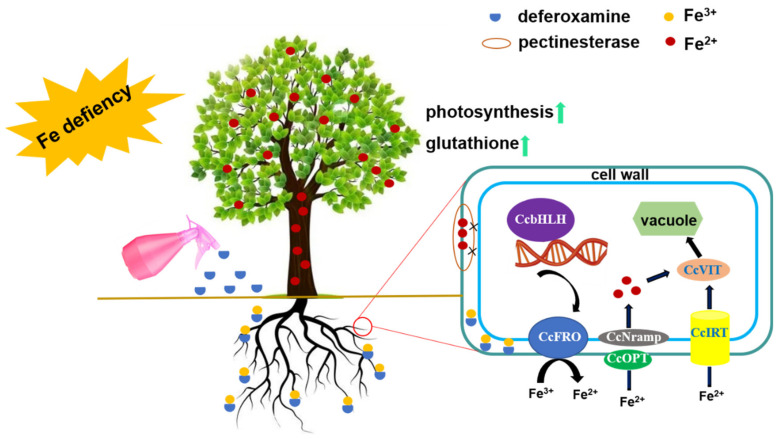
A schematic model for deferoxamine-alleviated iron deficiency in *C. camphora* plants. Supplemental DFO combined with insoluble Fe^3+^ in the soil form a complex. On the one hand, the root of *C. camphora* reduces Fe^3+^ in the complex to Fe^2+^ by *CcFRO* and then transports it to the nucleus by *CcNramp*, *CcOPT* and *CcIRT*. On the other hand, DFO improves the photosynthetic efficiency and endogenous glutathione content of *C*. *camphora* by inhibiting the fixation of iron ions by pectinesterase in the plant cell wall. Finally, the phenomenon of iron deficiency chlorosis in *C. camphora* is alleviated.

As shown in Figure 8, the present results indicated that the role of DFO in the alleviation of Fe deficiency chlorosis included an increase in Fe accumulation in leaves by regulating FCR activities and Fe uptake− and transport−related gene expression levels, thereby promoting the photosynthesis and growth of *C. camphora* seedlings. The biosynthesis of glutathione and related gene expression also played a vital role in modulating the DFO-mediated alleviation of Fe deficiency in *C. camphora*. These findings identified important genes related to iron metabolism in *C. camphora* and could provide an essential background for mitigating the symptoms of Fe deficiency.

## Figures and Tables

**Table 1:** Primers used in RT–qPCR analysis.

Gene Name	Gene Function	Primers
*CcFRO6*	Ferric reduction oxidase 6	AATGCCACAATGACAATA TAAGAAGACCAATCACAAG
*CcFRO8*	Ferric reduction oxidase 8	CATCTCTATTGCTGAACTTA GCTTATACTGCCATACATT
*CcIRT2*	Fe^2+^ transport protein 2	AAGATGGAGAAGATGACA ACTGAGTGAACTACAATTC
*CcVIT3*	Vacuolar iron transported 3	GTTCGTCTCCGTCTACTC CTCTCTTTACCCTCTTCCTT
*CcVIT4*	Vacuolar iron transported 4	TCTCCGTCTACTCACAAT CTTCCTCACCCTCTCTAC
*CcFER*	Ferredoxin−NADP reductase	TCTCCGTCTACTCACAAT CTTCCTCACCCTCTCTAC
*CcOPT3*	Oligopeptide transported 3	GGTCCTCTATTCTCAATC CGTGTCCATTAGAAGTAA
*CcNramp5*	Metal transporter 5	AACATCTATTATCTTAGCA TATCTATGAAGGTGACTA
*CcAHA2*	V−type proton ATPase 2	AGAAGAAGAAGAAGAAGACACT
		ATACATCTGCGTCGTTCAT
*CcPME*	Pectinesterase	TTGTAGGAGATGGAAGAGA
		GACTGTTGCTGAGTTGAA
*CcPMEI*	Pectinesterase inhibitor	CAGTTATGATGAGAAGGA
		ATTAGAGGAGGAGAAGAA
*CcEF1α*	Endogenous control, Reference gene	TCCAAGGCACGGTATGAT CCTGAAGAGGGAGACGAA

## Data Availability

All the data and materials have been provided in the main manuscript.
